# Pharmacokinetics of *Myo*-Inositol in a Wistar Rat Animal Model

**DOI:** 10.3390/ijms231911246

**Published:** 2022-09-24

**Authors:** Tomasz Antonowski, Adam Osowski, Damian Szczesny, Joanna Szablińska-Piernik, Jerzy Juśkiewicz, Lesław Lahuta, Andrzej Rynkiewicz, Joanna Wojtkiewicz

**Affiliations:** 1 Department of Human Physiology and Pathophysiology, School of Medicine, Collegium Medicum, University of Warmia and Mazury, 10-082 Olsztyn, Poland; 2 Department of Biopharmacy and Pharmacodynamics, Division of Pharmacodynamics, Medical University of Gdańsk, 80-210 Gdańsk, Poland; 3 Department of Plant Physiology, Genetics and Biotechnology, Faculty of Biology and Biotechnology, University of Warmia and Mazury, 10-719 Olsztyn, Poland; 4 Institute of Animal Reproduction and Food Research of Polish Academy of Sciences, 10-748 Olsztyn, Poland; 5 Department of Cardiology and Internal Medicine, School of Medicine, University of Warmia and Mazury, 10-045 Olsztyn, Poland

**Keywords:** myo-inositol, pharmacokinetic, Wistar rats

## Abstract

*Myo*-inositol is the most popular inositol in living organisms, where it is present as a sugar alcohol, in a free form, and can also be described as a lipid. The aim of this study was to check the concentration change of a myo-inositol solution from the time of oral administration and over a 48 h period in Wistar-type rats. All rats received 2 g/kg of inositol as a solution in distilled water by oral gavage. Estimated parameters were based on the serum concentration of myo-inositol observed in nine individual rats with regard to time. Observations were described as a one-compartment pharmacokinetic model with first-order absorption and zero-order endogenous input of checked inositol. The highest myo-inositol concentration was observed in the first hour after oral administration in the serum of all tested rats. Then, the concentration began decreasing immediately after the maximal peak. The inositol concentration continued to decrease, but after 24 h its level was still higher than before the administration. The plasma profile of the myo-inositol concentration showed a rapid decline over time, possibly due to the metabolism of this compound.

## 1. Introduction

Pharmacokinetics (PK) is the field of study of the time course of drug absorption, distribution, metabolism, and excretion of xenobiotic compounds such as new drugs within the body after their administration. This study is important for investigating the pharmacological efficacy and/or disposition profiles of all drug compounds in the body and may be influenced by clinical conditions such as overall age, gender or medical condition. Years ago, drug discovery research programs were usually focused on selecting the most potent and effective compound with the use of in vitro receptor binding tests or functional assays [1]. Safety and efficiency of natural compounds with potential bioactive properties are crucial. Currently, in the process of new drug development, pharmacokinetic methods are widely used in the verification of absorption, distribution, biotransformation and elimination of tested compound in animal and human models [2]. Absorption and distribution indicate the passage of the tested compound, such as drug molecules, from the moment of the administration [3]. Studies on laboratory animals may give useful information about drug development because bioactive compound levels in plasma or tissues are often easier to prepare and the results can be related to humans [4].

**Figure 1:**
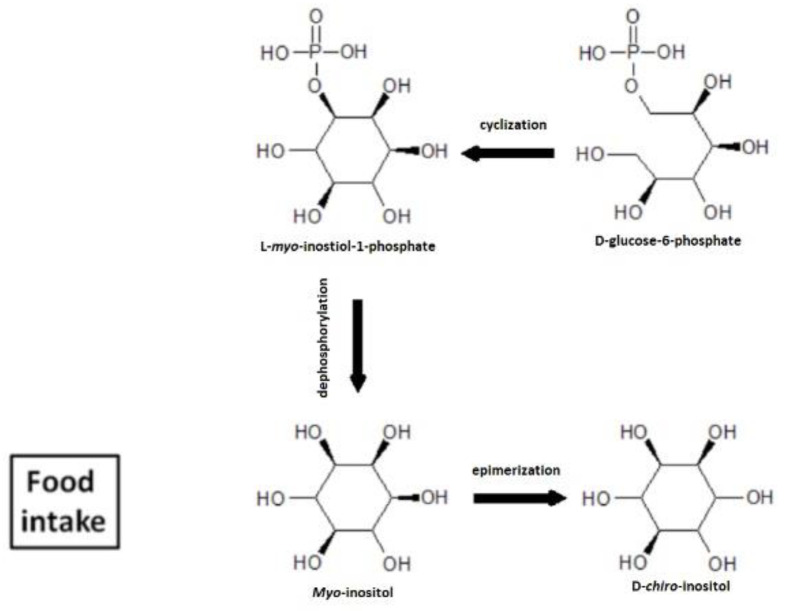
Cyclization, dephosphorylation and epimerization of MI.

Myo-inositol (MI) is the most popular inositol isoform. It is ubiquitous in living organisms, where it can be present in the form of a free sugar alcohol and as a group of membrane lipids. In the free form, inositol is a sugar alcohol available in the diet [5]. Its properties are similar to insulin; it acts as a second messenger in the intracellular insulin pathway and is involved in increasing sensitivity to insulin in various tissues, which improves metabolism and increases cellular glucose uptake [6,7]. Inositol is mainly catabolized by the liver, although it is excreted with urine only in small amounts [8,9]. It is worth mentioning that MI is enzymatically converted to D-chiro-inositol by the NADH-dependent epimerase, as shown in Figure 1 [10,11]. In the last two decades, MI has gained popularity in clinical reproductive practice, counteracting some types of tumors and metabolic disorders such as diabetes mellitus type II, polycystic ovary syndrome, thyroid disorders and infertility [12,13,14].

The aim of this study was to observe the changes in MI concentration from the time of administration and over 48 h. The Wistar rat animal model was used in this research.

## 2. Results

**Figure 2:**
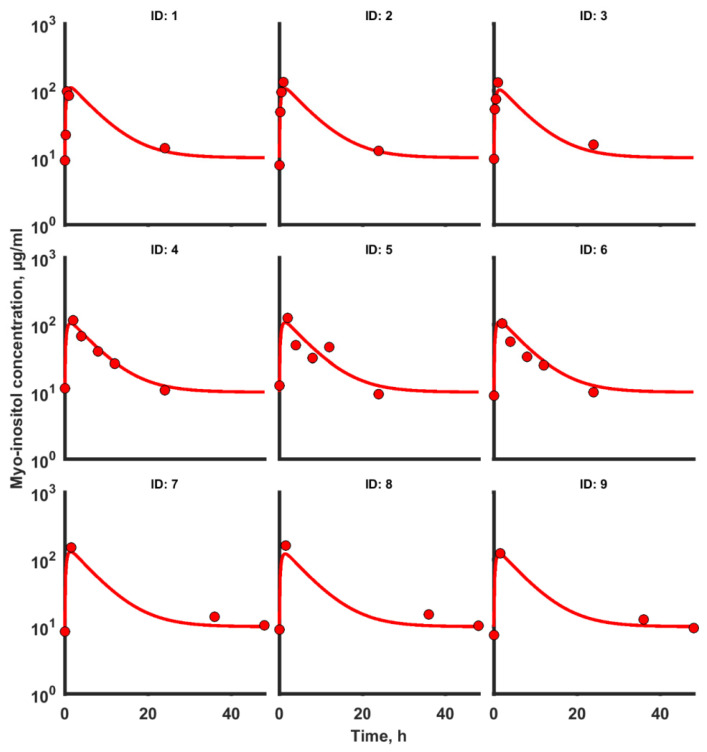
Plot of observed (circles) and individual predicted (solid lines) myo-inositol concentration–time profiles.

Estimated parameters were based on serum concentration–time profiles of myo-inositol observed in nine individual rats. Observations were best described by a one-compartment pharmacokinetic model with first-order absorption and zero-order endogenous input of myo-inositol. PK data, along with model predictions, are presented in Figure 2. The model describes the measured concentrations well.

The parameter estimates are listed in Table 1. All parameters were estimated with high precision (coefficient of variation lower than 15%). Due to a lack of data, some PK parameters (*CL* and *V*) are estimated as apparent.

**Figure 3:**
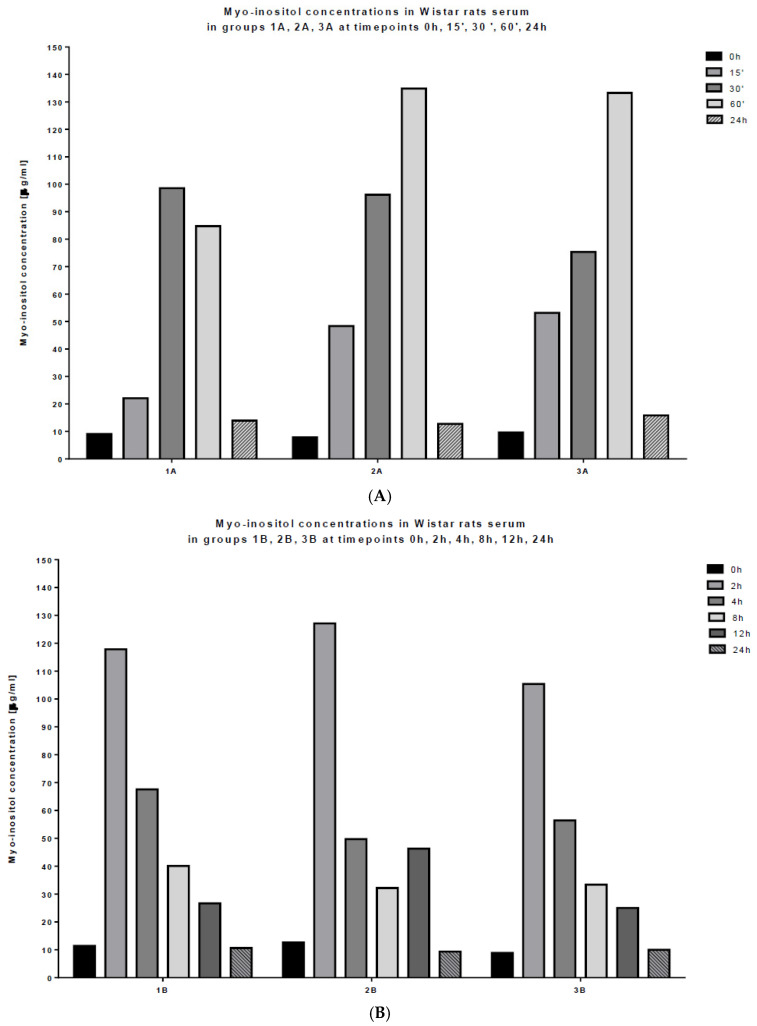

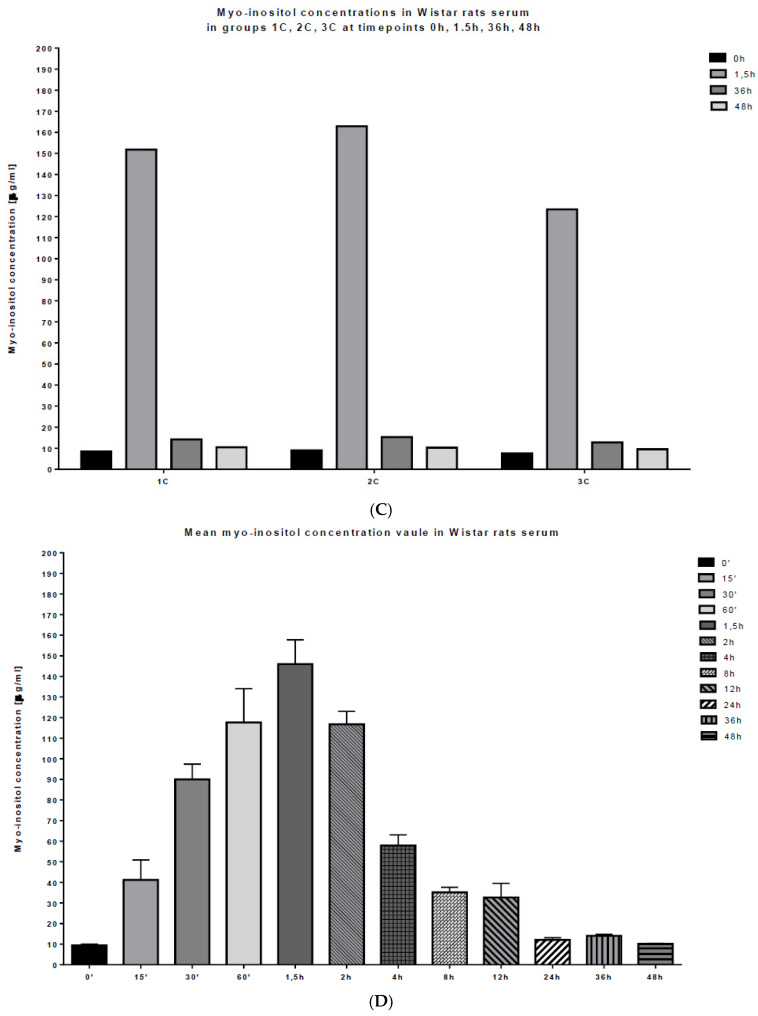

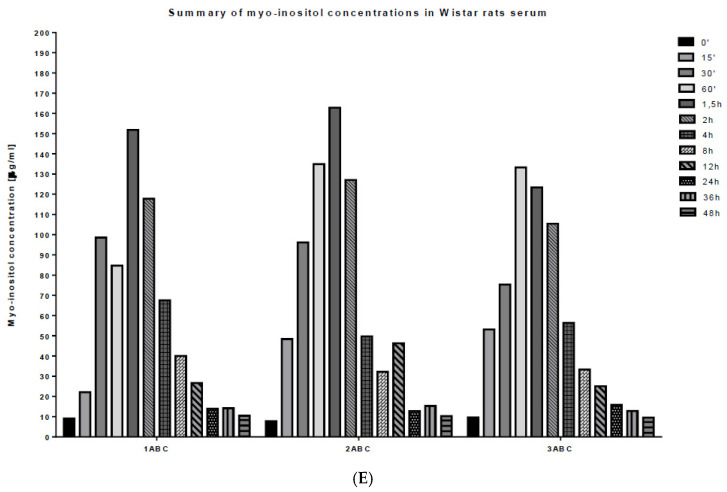
Myo-inositol concentrations in Wistar rat serum: (**A**) in groups 1A, 2A and 3A at time-points 0 h, 15′, 30′, 60′, 24 h; (**B**) in groups 1B, 2B, 3B at time-points 0 h, 2 h, 4 h, 8 h, 12 h, 24 h; (**C**) in groups 1C, 2C, 3C at time-points 0 h, 1.5 h, 36 h, 48 h; (**D**) summary of concentrations in all time-points; (**E**) mean concentrations in all time-points.

The highest mean myo-inositol concentration in serum was observed in the first hour after application in all nine of the tested rats (Figure 2 and Figure 3A–D). However, a decrease in the myo-inositol concentration started immediately after achieving the maximal peak. The concentration continued to decrease but, approximately after 24 h, its level was still higher than before administration. The plasma myo-inositol concentration over time profile showed a rapid decline, possibly due to the metabolism of the compound. Mean myo-inositol concentrations in serum for all treatment groups are shown in Table 2 and Figure 3E.

## 3. Discussion

Myo-inositol and its derivatives, such as D-chiro-inositol, constitute a large group of natural compounds. It is not only one of the most important membrane-incorporated phosphatidylinositols, but it acts as a free form, isomer or phosphate derivatives in many cellular processes such as: keeping metabolic homeostasis, mRNA export and translation, ion channel permeability, cytoskeleton remodeling and stress response [14,15].

In modern medicine, the most important features of a biologically active compound are bioavailability, safety, uptake and metabolism. These properties are widely discussed because of the complexity of the interconnected metabolic pathways. Besides being a structural element, myo-inositol has mostly unknown functions. However, several reports indicate that it may play an important role during the developmental period and during phenotypic transitions [15]. Furthermore, there is evidence that dysfunctions in the regulation of metabolism of this compound can be related to the appearance of several chronic diseases. Many clinical trials have proven that the use of inositol in pharmacological doses can generate positive results in the treatment of many diseases such as respiratory stress syndrome, Alzheimer’s disease, metabolic syndrome, gynecological diseases and, in some cases, tumors [15]. However, despite the widespread studies carried out to identify inositol-based effects, no comprehensive understanding of inositol-based mechanisms has been achieved [16,17].

In our study, MI at a 2 g/kg dose was effective in elevating serum levels and, importantly, these levels, in all tested rats, continued to stay elevated over time. Parameter estimation was based on serum concentration–time profiles of the tested compound observed in the animal models (Figure 3). Observations were best described by using the one-compartment disposition model with first-order absorption and zero-order input rates of myo-inositol. This was a single-compartment model that included factors for exogenous inositol level and allometric size based on weight. There was no significant evidence of harm from the dosage during the study, but future studies should monitor for possible adverse symptoms.

A similar study was conducted by Lennernäs et al. In that study, the disposition and emphasis on metabolism of A-trinositol was studied in rats. The drug was at a dose of 18.2 mmol/kg and was administered by intravenous bolus. It was followed by a short-term intravenous infusion of a-trinositol at 48.8 mmol/kg/h, which was administrated over 1.5 h. In the plasma samples, unchanged a-trinositol, inositol and water were detected, which indicates that, during the metabolism of a-trinositol, the main metabolites are formed. The plasma concentration profile over time of a-trinositol showed a very fast decline following the cessation of the intravenous infusion, which was most likely caused by the rapid metabolism of this compound in rats. The authors also examined a-trinositol metabolism with alkaline phosphatases from bovine intestinal kidney, liver and mucosa. They showed that intestinal enzymes cause extensive dephosphorylation of this compound [18]. In our study, we obtained similar results despite the difference in structure, administration and dose of the tested compound differences in the structure (A-trinositol has four rings in its structure compared to MI).

In another study, which was conducted by Moreira et al. in 2020, L-(+)-bornesitol’s (LB) pharmacokinetics were tested. LB also belongs to the cyclitol family. LB was isolated and purified from an ethanol extract of *Hancornia speciosa* leaves. *H. speciosa* is a medicinal plant with proven antihypertensive activity, whereas LB is the main compound of its leaves and is a potent angiotensin-converting enzyme inhibitor [19]. The authors investigated the pharmacokinetic properties of LB administered orally to the Wistar rats. Pharmacokinetics were evaluated by the administration of single doses: 3 mg/kg (via intravenous by bolus) and 3, 15 and 25 mg/kg (by gavage). Rat plasma was tested by using ultra-high performance liquid chromatography coupled with electrospray ionization mass spectrometry. LB reached peak plasma concentration within approximately 1 h after oral administration (with a half-life ranging from 72.15 to 123.69 min). The LB’s peak concentration and area under the concentration time curve did not rise proportionally with the increasing doses, which showed non-linear pharmacokinetics in rats. The same research group had previously demonstrated that LB administered to Wistar rats at 3 mg/kg reduced the systolic blood pressure by a mechanism resulting from an increase in NO production and ACE inhibition. For this reason, the same dose was initially given as intravenous bolus and orally to the animals in order to correlate the pharmacological response. Additionally, increasing doses were also administered (15 mg/kg and 25 mg/kg) to check whether LB follows a linear pharmacokinetic. After administration, LB was rapidly distributed, which was followed by a slow elimination process. These data suggest the presence of a peripheral compartment, which is consistent with the elevated volumes of distribution [19]. In our experiment, we obtained a similar result, but the level of MI in the serum of rats began to drop significantly only 2 h after oral administration. This difference is most likely due to the doses in which both tested compounds were administered: MI was administered at a dose of 2 g/kg, while the highest dose of bornesitol was 25 mg/kg. Additionally, it should be considered that, chemically, bornesitol is a methyl ether of D-myo-inositol (it has an additional methyl group instead of hydrogen on one hydroxyl group). The presence of this additional methyl group may be responsible for a more rapid metabolism of this compound in cells.

In the study conducted by Navaro et al. in 2020, the authors showed the new metabolic effect of D-pinitol (DP), a natural inositol from carob fruit. To characterize the metabolic actions of this dietary inositol in male Wistar rats, the authors analyzed its pharmacokinetics. Oral DP administration in doses of 100 and 500 mg/kg resulted in its rapid absorption and distribution to plasma. Pharmacokinetic analysis showed a rapid absorption of DP in the plasma and liver of Wistar Rats after an acute oral load. This has been obtained by monitoring the plasma concentration at 0, 10, 20, 30, 60, 120, 240 and 360 min after the oral administration of 100 mg/kg in 18 h food-deprived male Wistar rats. The authors showed that, after supplementation, plasma DP level became detectable after 10 min, and peaked at 1 h, showing its rapid absorption and clearance. The liver concentration of DP became detectable at 30 min after oral administration. A peak liver concentration of DP was observed at 120 min. It should be mentioned that no accumulation was detected in the liver due to a rapid clearance of the studied compound from the hepatic tissue. In higher oral doses (500 mg/kg), DP peaked at 120 min both in plasma and liver but still showed a rapid clearance. To sum up, the pharmacokinetic analysis showed that the oral DP is absorbed and quickly detected in plasma and liver, reaching a peak in 1 h to be completely cleared after 6 h. Liver accumulation of this inositol was shifted in time by 1 h with a similar rate of clearance. Thus, the half-life time for plasma and liver was 108 min and 154 min, respectively. Similar results were observed with the highest peak of MI in rat plasma at 1 h after administration; this is most likely caused by the difference in the doses of both tested compounds (MI was administrated as a 2 g/kg dose, DP was administrated in 100 and 500 mg/kg doses). In the article by Navaro et al. in 2020, the level of DP in rat plasma rapidly decreased around 1 h after administration. In our study, the level of MI started to drop after 2 h [20].

Based on our observations, we cannot conclude that myo-inositol did not cause any undesirable metabolic changes in rats.

## 4. Materials and Methods

### 4.1. Animals and Study Design

The experiment was performed on male Wistar rats (n = 9). We used male Wistar rats in our study because hormone changes throughout the female estrus cycle may have caused female rats to react differently, making them unpredictable and providing variable results during repeated experiments. All rats were 6 weeks old and had no morphological features deviating from the standard Wistar animal model. Animals were housed in a temperature-controlled room with a 12 h light/dark cycle and free access to water and food. The study was conducted in compliance with the Local Ethics Committee for Animal Experiments in Olsztyn. Each animal received a solution of myo-inositol (2 g/kg) in distilled water by oral gavage (Agnthos, curved gastric probe, 16G, 3 mm, 75 mm tip). The animals were fasted before the administration of myo-inositol (12 h since the last meal). Food was allowed for 2 h after administration of the compound. Access to water was unlimited. In order to reduce the amount of blood collected from a single animal, the animals were divided into three groups (n = 3). Each group had blood collected at different time points as indicated in Table 2.

### 4.2. Analysis of Myo-Inositol in Rat Serum by GC Method

For removing of proteins, 160 μL of acetonitrile (containing 100 g of xylitol, as an internal standard) was added to 50 μL of rat plasma and the sample was shaken for 5 min (1300 rpm, Vortex Genie 2, Scientific Industries, Bohemia, NY, USA). After centrifugation (20,000× *g* at 4 °C for 10 min), the supernatant was transferred into glass chromatographic vials and concentrated in a speed vacuum evaporator to dryness. Dry residues were derivatized with a mixture of trimethylsilylimidazole (TMSI) and pyridine (1:1, *v*/*v*). TMS-derivatives of myo-inositol were analyzed by the high-resolution gas chromatography method on a gas chromatograph GC2010Plus (Shimadzu, Tokyo, Japan) with a capillary column ZEBRON ZB-1 (15 m length, 0.25 mm diameter and 0.1 μm film thickness, Phenomenex, Torrance, CA, USA). The injector temperature was 325 °C and the initial column oven temperature was 150 °C. Helium was used as a carrier gas (at a flow rate of 1.18 mL min^−1^). Afterwards, the sample injection (1 μL, in a split ratio 10:1) was ramped sequentially to 200 °C at a rate of 20 °C min^−1^, to 300 °C at 30 °C min^−1^ and to 335 °C at 20 °C min^−1^. The final temperature was held for 2.42 min and the total time of analysis was 10 min. The detector was maintained at 350 °C. Myo-inositol was quantified by using the standard: *myo*-inositol from Sigma. The content of myo-inositol was calculated from the standard curves of appropriate components.

### 4.3. Analytical Method Description—PK Model

**Figure 4:**
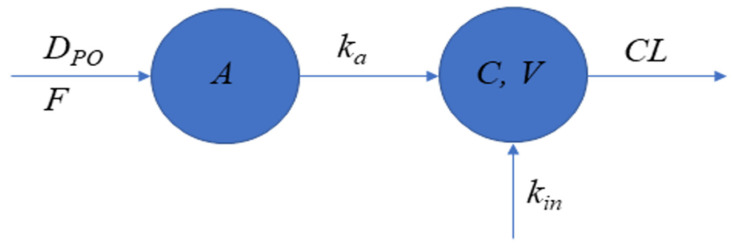
Graphic representation of PK model for myo-inositol. The serum concentration of myo-inositol was described by a one-compartment disposition model with first-order absorption and zero-order input rates.

The data fitting was performed with NONMEM software (Version 7.3.0; ICON Development Solutions, Ellicot City, MD, USA) and the Gfortran compiler 9.0. NONMEM runs were executed using Wings for NONMEM (WFN730; http://wfn.sourceforge.net). The NONMEM data processing and plots were performed in Matlab Software (Version 0.1; The MathWorks, Natick, MA, USA). The schematic representation of the PK model is given in Figure 4.

$$\frac{dA}{dt}=-k_{a}*AA\left(0\right)=D_{PO}$$

$$\frac{dX}{dt}=k_{}-\frac{CL/F}{V/F}*A+k_{a}*AX\left(0\right)=C_{0}*V/F$$

where *A* and *X* denote the amount of myo-inositol in the absorption and central compartment, *F* denotes bioavailability, *D_PO_* denotes an oral dose of myo-inositol, *k_a_* denotes absorption rate constant, *k_in_* denotes an endogenous input of myo-inositol, *CL* denotes clearance, *V* denotes volume of distribution, and *C*_0_ denotes endogenous concentration of myo-inositol. *CL* and *V* were estimated as apparent due to uncertain bioavailability (*CL/F* and *V/F*). The steady-state concentration of myo-inositol implies the following relationship:

$$k_{}=C_{0}*CL/F$$

The concentration of myo-inositol in serum is given by:

$$C=\frac{X}{V/F}$$

The observed concentrations (*C_obs_*) were related to model predictions through the following equation (proportional residual error model):

$$C_{obs}=C*\left(1+\varepsilon \right)$$

where ε is a normal random variable with standard deviation σ.

## 5. Conclusions

We have shown that myo-inositol administered via an oral pathway to rats is almost completely metabolized over time. Inositol metabolism is complex and not fully understood. Its serum concentration changes over time, which may be influenced by the supply of this compound along with nutrition and complicated endogenous controls taking place inside the body.

## Figures and Tables

**Table 1:** Summary of the final PK parameters and residual error variance estimates of myo-inositol.

Parameter, Unit	Description	Estimate (% CV)
*V/F* [L]	Apparent volume of central compartment	5.1 (6.5)
*CL/F* [L/h]	Apparent elimination clearance	0.853 (10.1)
*K_a_* [1/h]	Absorption rate constant	1.89 (10.7)
*C*_0_ [mg/L]	Concentration of myo-inositol at t = 0	10 (4.0)
**Residual variability**		
σ^2^ (%)	Proportional residual error variability	24.3 (11.6)
**Secondary parameters**		
*K_el_* [1/h]	Elimination rate constant, (CL/F)/(V/F)	0.17
*T*_0.5_ [h]	Biological half-life, ln(2)/k_el_	4.08
*k_in_* [1/h]	Endogenous input of myo-inositol, C_0_·CL/F	8.53
AUC [mg/L·h/kg]	Area under concentration–time curve, F·D/CL	2.34

**Table 2:** Detailed information about animals and study design. Blood samples in the volume of 200–300 µL were collected from the tail and centrifuged; the serum was then collected and stored at −80 °C until further analysis.

Group	Time [h]	Rat ID	Body Weight [g]	Mean MI in Rat Serum [µg/mL]
**1**	0; 0.25; 0.5; 1; 24	1A (ID:1)	328	45.69
		2A (ID:2)	316	60.00
		3A (ID:3)	302	57.46
**2**	0; 2; 4; 8; 12; 24	1B (ID:4)	309	45.70
		2B (ID:5)	317	46.23
		3B (ID:6)	330	39.86
**3**	0; 1.5; 36; 48	1C (ID:7)	391	46.23
		2C (ID:8)	361	49.38
		3C (ID:9)	359	38.33

## Data Availability

Not applicable.
